# Combination antiretroviral drugs in PLGA nanoparticle for HIV-1

**DOI:** 10.1186/1471-2334-9-198

**Published:** 2009-12-09

**Authors:** Christopher J Destache, Todd Belgum, Keith Christensen, Annemarie Shibata, Akhilesh Sharma, Alekha Dash

**Affiliations:** 1 Department of Pharmacy Practice, Creighton University School of Pharmacy & Health Professions, Omaha, NE, USA; 2 Department of Biology, Creighton University, Omaha, NE, USA; 3 Department of Internal Medicine, Creighton University School of Medicine, Omaha, NE, USA; 4 Department of Pharmacy Sciences, Creighton University School of Pharmacy & Health Professions, Omaha, NE, USA

## Abstract

**Background:**

Combination antiretroviral (AR) therapy continues to be the mainstay for HIV treatment. However, antiretroviral drug nonadherence can lead to the development of resistance and treatment failure. We have designed nanoparticles (NP) that contain three AR drugs and characterized the size, shape, and surface charge. Additionally, we investigated the *in vitro *release of the AR drugs from the NP using peripheral blood mononuclear cells (PBMCs).

**Methods:**

Poly-(lactic-co-glycolic acid) (PLGA) nanoparticles (NPs) containing ritonavir (RTV), lopinavir (LPV), and efavirenz (EFV) were fabricated using multiple emulsion-solvent evaporation procedure. The nanoparticles were characterized by electron microscopy and zeta potential for size, shape, and charge. The intracellular concentration of AR drugs was determined over 28 days from NPs incubated with PBMCs. Macrophages were imaged by fluorescent microscopy and flow cytometry after incubation with fluorescent NPs. Finally, macrophage cytotoxicity was determined by MTT assay.

**Results:**

Nanoparticle size averaged 262 ± 83.9 nm and zeta potential -11.4 ± 2.4. AR loading averaged 4% (w/v). Antiretroviral drug levels were determined in PBMCs after 100 μg of NP in 75 μL PBS was added to media. Intracellular peak AR levels from NPs (day 4) were RTV 2.5 ± 1.1; LPV 4.1 ± 2.0; and EFV 10.6 ± 2.7 μg and continued until day 28 (all AR ≥ 0.9 μg). Free drugs (25 μg of each drug in 25 μL ethanol) added to PBMCs served as control were eliminated by 2 days. Fluorescence microscopy and flow cytometry demonstrated phagocytosis of NP into monocytes-derived macrophages (MDMs). Cellular MTT assay performed on MDMs demonstrated that NPs are not significantly cytotoxic.

**Conclusion:**

These results demonstrated AR NPs could be fabricated containing three antiretroviral drugs (RTV, LPV, EFV). Sustained release of AR from PLGA NP show high drug levels in PBMCs until day 28 without cytotoxicity.

## Background

An estimated 36 million people are infected with human immunodeficiency type-1 (HIV-1) worldwide [1]. The majority of infected people live in the developing world with limited treatment resources. Antiretroviral (AR) therapy has significantly reduced HIV-1 disease morbidity and improved life expectancy. However, the economics of drug treatment, treatment failures due to the development of resistance, and limited global access has prevented world-wide utility of AR therapy [2,3]. Dosing regimens that require multiple daily dosing with diet considerations and AR side effects have compromised the achievement of long-term HIV-1 suppression in infected patients [4]. Additionally, the use of AR requires a concerted level of commitment from the patient to prevent treatment failure due to resistance.

The CD4^+ ^T lymphocyte is the major target for infection by HIV-1. Cells of the mononuclear phagocyte system also serve as a reservoir for HIV. Macrophages are a mature, non-proliferating and immunologically active cells that can be productively infected with HIV-1 and HIV-2 [5-8]. Altered cellular functions in the macrophage population may contribute to the development and clinical progression of AIDS.

Evidence has accumulated that cells of the macrophage lineage are vectors for the transmission of HIV-1. The placental macrophage is likely to be the primary cell type responsible for vertical transmission of HIV-1 [9]. An important property of HIV-1 for mucosal transmission is the ability to infect macrophages [10]. Because of the important role of cells of the monocytes/macrophage (Mo/Mac) lineage in the pathogenesis of HIV-1, fully effective AR must react with Mo/Mac in addition to other targets.

Many promising compounds suffer from poor physiochemical properties leading to poor solubility and biodistribution. Such properties limit drug-receptor interactions to cause desired effects. For example, proteins and peptides could be new drug candidates but suffer from low oral absorption in the gastrointestinal tract and necessitate daily subcutaneous administration. Nanoparticles may be able to be administered parenterally but possibly not daily. New drug candidates must demonstrate that they reach the site of action and have a pharmacologic effect. Special drug carrier systems and dosage forms such as nanoparticles, hold the promise of overcoming obstacles to bring about successful therapy [11]. Nanoparticles are stable, solid colloidal particles consisting of macromolecular material ranging in size from 10 to 1,000 nm. Drugs can be adsorbed on the particle surface, entrapped within the particle, or dissolved in the particle matrix [12,13]. Nanoparticles represent an interesting carrier system for the transport of antiviral drugs to Mo/Mac in an attempt to reduce the required dose, minimize toxicity and side effects, and improve the delivery of substances, which have insufficient intracellular uptake.

Previous studies have demonstrated that AR can be adsorbed onto a nanoparticle or microparticle carrier [14-20]. These studies show that one antiretroviral drug can be fabricated into nanoparticles as a drug delivery system. This report shows the *in vitro *effect of fabricating nanoparticles that contain ritonavir, lopinavir, and efavirenz for sustained drug delivery. To the best of our knowledge, this is the first study that attempts to fabricate three antiretroviral drugs in a polymeric nanoparticulate system.

## Methods

### Nanoparticle (NP) preparation

AR (ritonavir, lopinavir, efavirenz) was prepared using a water-in-oil-in-water homogenization. Briefly, in a typical procedure, AR drug powder (5 mg of each AR drug) was added to poly-(lactic-co-glycolic acid) (PLGA) polymer (molecular weight 110,000-139,000 Daltons (50 mg)) in 10 mL methylene chloride. The solution was heated in an incubating shaker at 36°C and slowly stirred for a minimum of 40-45 minutes. After the PLGA polymer was dissolved, it was drawn into a glass syringe and added in drop-wise fashion to a solution of 0.25% w/v aqueous solution of polyvinyl alcohol (PVA). A probe sonicator (100 W for 6 min) (Sonicator XL, Misonix, Farmingdale, NY) was used to homogenize the emulsion. The water-in-oil emulsion that was formed was then placed in a glass syringe and added to 30 mL of ethylene oxide/propylene oxide block copolymer (Poloxamer-127; 2% [w/v] 30 mL); BASF, Mt. Olive, NJ) while the block copolymer was being homogenized by the probe homogenizer as described above for 15 minutes to form the water-in-oil-in-water emulsion.

NPs containing osmium tetroxide, an electron-dense agent, were formulated similarly, except that 10 mg of osmium tetroxide, and one milligram of each AR was added to the polymer solution and the procedure was followed as above. Additionally, 6-hydroxycoumarin (a fluorescent dye; 100 μg) was added to the polymer solution and one milligram of each AR was fabricated to make fluorescent NPs for flow cytometry and the procedure was followed as above. In all formulation procedures, the emulsion that was formed was stirred for a minimum of 4 hours at room temperature to evaporate the organic solvent. The particles were rinsed twice with ddH_2_O to remove PVA and unentrapped drugs followed by ultracentrifugation (15,000 G for 45 minutes at 4°C, Optima LE-80K, Beckman, Palo Alto, CA), and then lyophilized (-52°C and 5.62 torr; Labconco, Freezone 4.5) for 24 hours to obtain a dry powder.

### Nanoparticle characterization

Nanoparticles were evaluated for size by zeta potential and confirmed by scanning electron microscopy (SEM) and surface change by using a zeta potential analyzer (ZetaPlus, Brookhaven Instruments, Holtsville, NY). For SEM, an aliquot of the fabricated nanoparticles, was suspended in double-distilled water for a final concentration of 0.2 mg/ml and an aliquot of suspended particles were placed onto a scanning electron tip and sputter coated with 2% w/v uranyl acetate, dried, and then visualized by using a JEOL-40A (JEOL Ltd, Sheboygan, WI) scanning electron microscope.

### High-pressure liquid chromatography (HPLC)

HPLC was performed using a previously reported method [21]. Briefly, the equipment included a pump (LC-10ATvp), system controller (SIL-10ADvp); degasser unit (DGU-14A), refrigerated auto-sampler (SIL-10ADvp); and a UV-Vis detector (SPD-10ADvp) and a column heater (set at 35°C) (all from Shimadzu Corporation, Columbia, MD). Samples were run through a C_18 _pre-column and a Jupiter C_18 _reverse-phase [150 × 3.9 mm (I.D.)] with 5 mm particle size packing (Phenomenex, Torrance, CA). The mobile phase was 25 mM KHPO_4 _(pH 4.9) and acetonitrile (40:60). The mobile phase was filtered and degassed prior to use. Flow rate was set at 0.9 mL/min and the detector was set at 212 nm. Samples of known amounts of the AR drugs (lopinavir, ritonavir, and efavirenz) were diluted in methanol to obtain a 45-1000ng/ml standard curve and then further diluted in PBS. Peak area from the samples and standards were integrated using EZ-Start chromatography software (Shimadzu) on a Dell computer. Injection volume was 20 μL and all experimental samples were analyzed in duplicate and averaged. Standards were analyzed in triplicate and averaged. Triplicate experiments were performed and all results are reported as mean ± standard error of the mean (SEM). One milligram of formed particles was dissolved in one-milliliter methylene chloride to dissolve the NP, in glass tubes and evaporated overnight in quadruplicate. High-pressure liquid chromatography (HPLC) mobile phase (200 μL) reconstituted the tubes for 5 minutes, the tubes were vortexed for 1 minute. The tubes were centrifuged (11,000 rpm, 10 minutes, 4°C) and aliquots were injected into the HPLC equipment to determine AR drug loading, loading efficiency, and entrapment efficiency. Inter-day and intra-day variability was always < 10%.

### Human monocytes isolation and cultivation

Human PBMCs were obtained from whole blood collection of HIV-1, -2 and hepatitis B seronegative donor and purified using CPT Vacutainer tubes (B-D and Co., Sparks, MD) according to the manufacturer instructions. Polymorphonuclear cells (1 × 10^6 ^cells/mL) were cultured in DMEM supplemented with 10% heat-inactivated pooled human serum, 1% glutamine, 1% penicillin-streptomycin, and 10 μg/mL ciprofloxacin (Sigma Chemical Co) then filter sterilized. The PBMCs were used within 2 hours after blood collection. Cells were incubated at 37°C and 5% CO_2_. Media was one-half exchanged with fresh media every 2-3 days. These cells were used for AR drug release experiments from NPs as determined by HPLC.

Human PBMCs at 5 × 10^6 ^cells/mL were cultured in DMEM supplemented with 10% heat-inactivated pooled human serum, 1% glutamine, 1% penicillin-streptomycin, and 10 μg/mL ciprofloxacin (Sigma Chemical Co), and 1000 U/mL highly purified recombinant human macrophage colony stimulating factor (M-CSF; R&D Systems, Inc; Minneapolis, MN) for seven days to differentiate monocyte-derived macrophages(MDM)[22]. Media was one-half exchanged every 2-3 days. Monocyte-derived macrophages were used for TEM and fluorescent imaging.

### AR Release from Nanoparticles

Antiretroviral NPs (2 mg in 250 μL phosphate buffered saline (PBS)) were added to PBMC cell culture flasks and kept in the incubator. At specific times, media in the flask was removed and placed in a sterile 15 mL conical tube and centrifuged (400 × G, 24°C for 10 minutes) to pellet the cells and the media was removed. Cell samples were obtained every 2 hours for the first 8 hours, then 2, 3, 4, 6, 10, 14, 21, and 28 days. Cells (250 μL) were removed from the tube and put into a microfuge tube for HPLC analysis. Two hundred fifty microliters of 100% methanol was added to the microfuge tube to lyse the cells, vortexed for 1 minute and the cells were frozen (-20°C) until assayed for AR drugs using HPLC. When HPLC was performed, microfuge tubes were thawed, centrifuged at 15,000 rpm at 4°C for 10 minutes and an aliquot of supernatant was placed into autosampler vials with glass insert. Free drugs (25 μg/mL of each AR drug) was dissolved in HPLC-grade ethanol and then further diluted in PBS, incubated with the PBMCs and cells were removed at 4, 8, 24, and 48 hours, lysed with methanol, centrifuged, and assayed by HPLC as controls of these experiments.

### Electron microscopy

To determine the shape and size of AR nanoparticles, nanosuspensions were examined with a JEOL 40A scanning electron microscope. NP shape and structural integrity were examined in thin sections. For TEM, MDM were exposed to AR nanoparticles at 5 × 10^-6 ^M for 30 minutes and 1 hour. Cells were rinsed with PBS, fixed with 2.5% glutaraldehyde for 24 hours, post-fixed with 1% osmic acid, dehydrated in graded ethanol solutions, and embedded in Epon 812 mixture. Thin sections were cut and stained with 2% uranyl acetate and examined under a JEOL-1011.

### Analysis of fluorescent AR nanoparticle uptake

The ability of MDMs to uptake fluorescent AR nanoparticles was assessed using FLOW cytometry analysis and direct fluorescence microscopy. Chamber slides containing MDM (2.5 × 10^5^) previously cultured had 25 μg of fluorescent AR nanoparticles in 50 μL PBS added to each well for 30 and 60 minutes. MDMs cultured in the absence of nanoparticles were used as controls. Following incubation, cultured MDM were rinsed with PBS and fixed in 4% paraformaldehyde in PBS for 10 minutes and coverslipped. Cells were visualized with an inverted fluorescent microscope (DMI4000B, Leica) and images were acquired using Image ProPlus software (Media Cybernetics; Bethesda, MD).

FLOW cytometry analysis used control and MDMs incubated with fluorescent AR nanoparticles as above. These cells were scraped from 6 well culture plates following incubation, centrifuged for 2-4 minutes at 1,200 rpm, rinsed in phosphate buffered saline (PBS), and fixed in 10% buffered formalin. Fixed cells were resuspended and samples were run on a UV SORP FACSAria (BD Biosciences, San Jose, CA). FLOW used 100 mW Coherent Sapphire laser set at 488 nm for excitation and was detected using a 530/30 bandpass filter (looking at light between 515 nm and 545 nm).

### Analysis of macrophage viability

MDM viability following exposure to and phagocytosis of nanoparticles was measured by using the MTT (3-(4,5-dimethylthiazol-2-yl)-2,5-diphenyltetrazolium bromide) method [23]. Active mitochondrial dehydrogenases in healthy cells convert MTT generating water-insoluble, purple formazan crystals that are measured by spectrophotometric techniques [24]. For each MTT assay, 2.5 × 10^5 ^differentiated human macrophages were plated on 24 well tissue culture plates using the media as previously described. Macrophages were incubated with or without AR nanoparticles immediately preceding and one hour prior to application of MTT. MDMs were allowed to metabolize MTT (5 mg/ml in DMEM supplemented media) for 30 or 60 min at 37°C and 5% CO_2_. Media was removed from cultured macrophages and cells were treated with 100% dimethyl sulfoxide to lyse the cells and dissolve formazan crystals. Lysates were transferred to 96 well plates for analysis. Absorbance of the lysate was measured at 595 nm using a precision microplate reader (Molecular Devices, model S/NE10984). Blank wells were subtracted as background from each triplicate sample and the samples were averaged.

## Results

**Figure 1:**
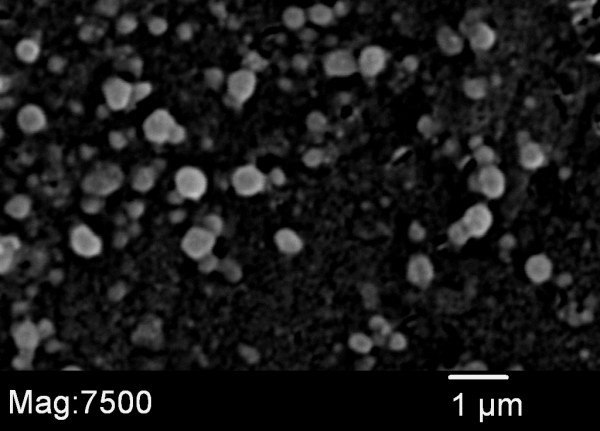
**Scanning electron microscopy (SEM) of fabricated antiretroviral nanoparticles (Mag × 7500)**.

Antiretroviral nanoparticle size and particle charge (n = 9) were measured after three batches. The particles averaged (± SEM) 262 ± 83.9 and -11.4 ± 2.4, respectively. Figure 1 depicts the SEM photomicrograph of AR nanoparticles. AR drugs were analyzed by HPLC for nanoparticle loading and loading efficiency. Antiretroviral drug loading averaged 4.9%, 5.2%, and 1.9% for RTV, LPV, and EFV, respectively. Entrapment efficiency averaged 38%, 45%, and 86% for RTV, LPV, and EFV, respectively.

**Figure 2:**
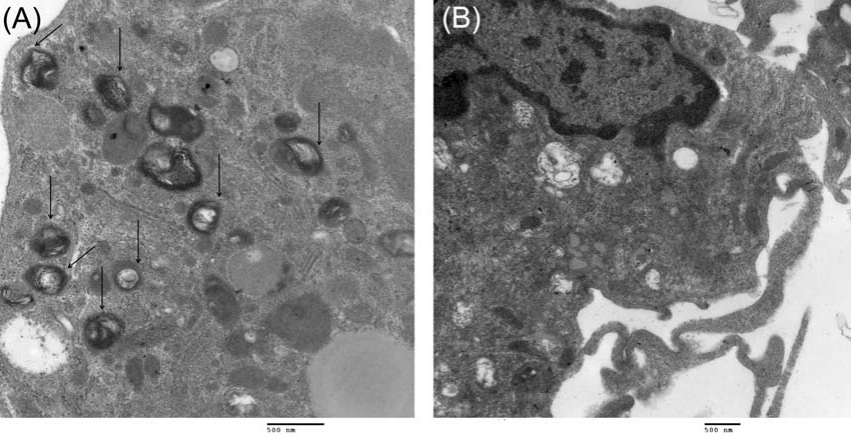
**Transmission electron microscopy (TEM) of NPs in MDM**. Transmission electron microscopy (TEM) of nanoparticles (NPs) within macrophages. Photos are high magnification of MDM containing ART NPs (arrows; A) and control MDMs (B) ladened with osmium tetroxide after 45 minutes of incubation. Mag × 40,000.

Osmium tetroxide ladened AR NPs were incubated with macrophages for 0, 0.5, and 1 hour. Figures 2 show TEM photomicrographs of osmium tetroxide ladened AR NPs within macrophages as well as AR NPs undergoing phagocytosis.

**Figure 3:**
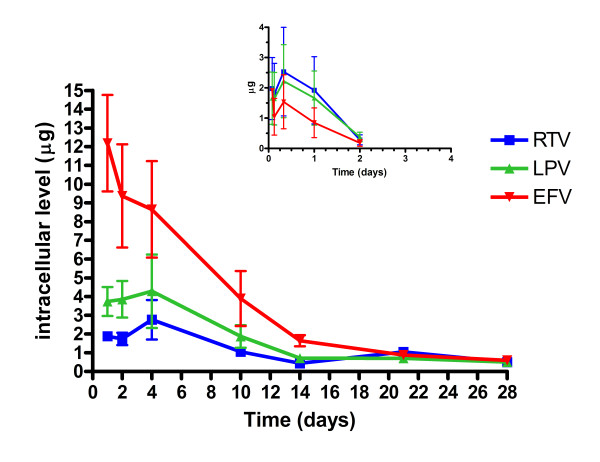
***In vitro *ART release from NPs incubated in PBMCs**. Intracellular ritonavir, lopinavir, and efavirenz levels in PBMCs over time. The insert figure is the intracellular free drug levels in PBMCs over time.

Antiretroviral drug release from PLGA NPs incubated with polymorphonuclear cells (PBMCs) is shown in Figure 3. The inset figure is the HPLC analysis of the free drug incubated with the PBMCs. Free drug incubated with PBMCs demonstrate removal of AR drugs by day 2 *in vitro*. When the cells were lysed and analyzed by HPLC, the free intracellular concentrations of the three drugs peaked at 8 hours (RTV 5.1 ± 0.05; LPV 4.3 ± .03; and EFV 3.1 ± 0.02 μg) and were eliminated by 48 hours. In contrast, when AR were fabricated into a NP and incubated in PBMCs, intracellular AR peak concentrations were at 96 hours (RTV 2.5 ± 1.1; LPV 4.1 ± 2.0 μg). Efavirenz intracellular concentration peaked at 24 hours (12.6 ± 2.7 μg). All three drugs continued to be released for 28 days. The 28 day concentrations for the three ARs were ≥ 0.9 μg.

**Figure 4:**
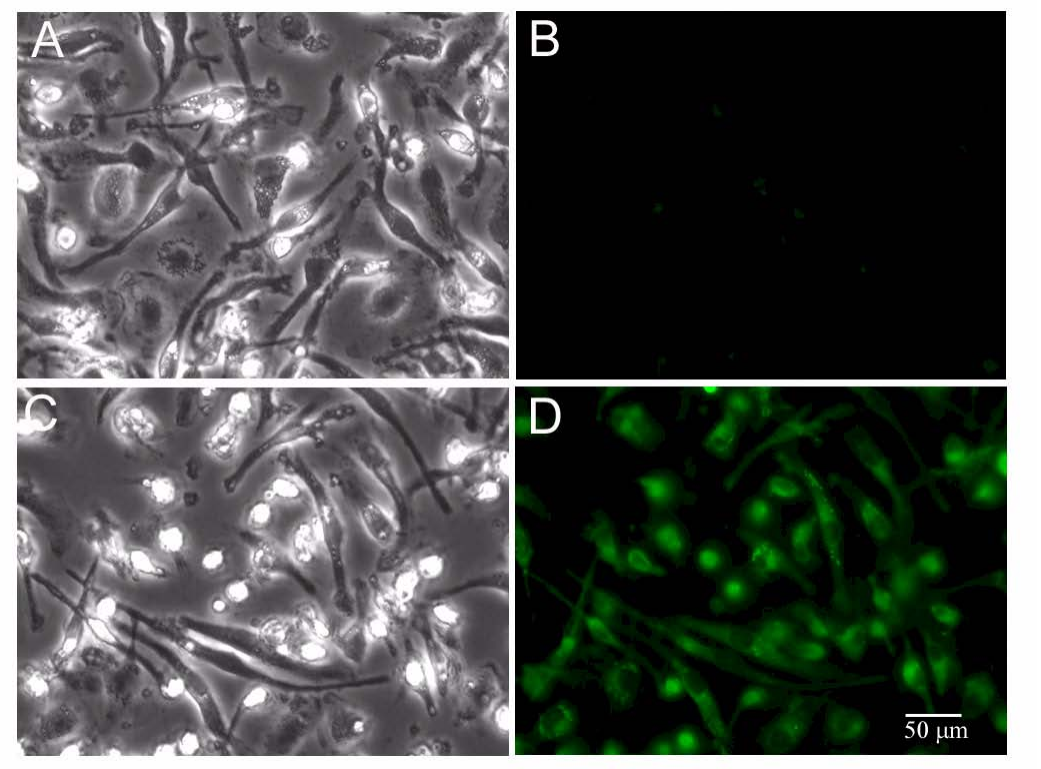
**Fluorescent NP uptake by human MDM**. Fluorescent NP uptake by human MDM. A and B are control phase and fluorescent photomicrographs of human MDM in the absence of fluorescent NPs. Following 30 min incubation with NPs, MDM fluoresce due to NP uptake (C and D; 40× objective).

6-Hydroxycoumarin was used to determine the efficiency with which macrophages phagocytize fluorescent NPs. Fluorescent macrophages were observed by FLOW cytometry as well as by fluorescent microscopy. FLOW cytometry data shows that virtually all macrophages phagocytized fluorescent NPs (data not shown). Direct fluorescence showed the presence and relative localization of nanoparticles in macrophages following incubation and uptake (Figure 4). While all imaged cells show uptake of the fluorescent AR NPs, fluorescence is not seen in control cells.

**Figure 5:**
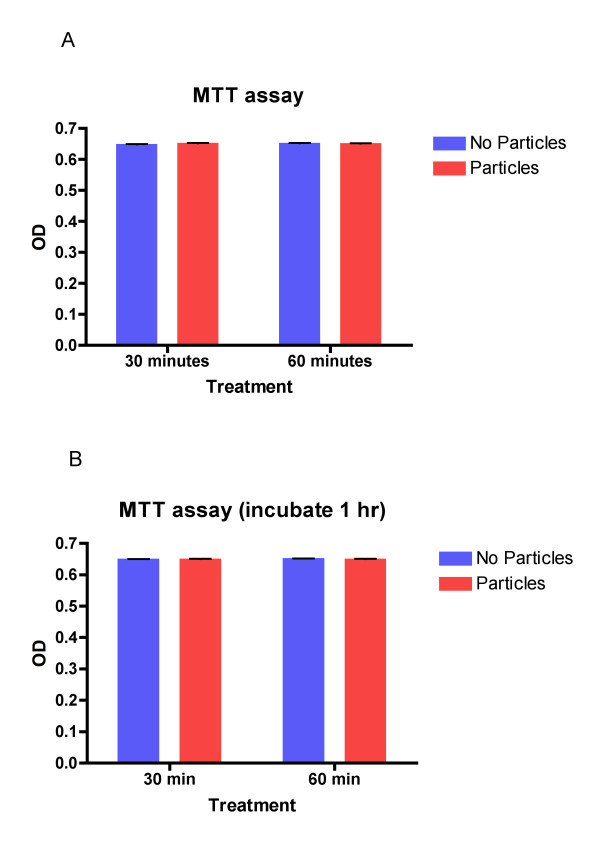
**MTT assay results**. Graphical representation of MTT assay for control macrophages (MDM) and MDMs incubated with nanoparticles. Nanoparticles and MTT substrate were immediately added to the media of cultured human MDMs MTT assays were performed after 30 and 60 minutes (Panel A) of incubation. Alteratively, MDMs were incubated with and without nanoparticles for 1 hour before the MTT substrate was added and an MTT assay was performed after 30 and 60 minutes (Panel B) of incubation.

To address whether the uptake of AR NPs by macrophages affected cell viability, MTT assays were performed (Figure 5). MTT assays measure the viability of cells by assessing the presence of active mitochondrial dehydrogenases that convert MTT into water-insoluble, purple formazan crystals. Solubilization and analysis of formazan conversion demonstrates that immediately following nanoparticle addition and one hour after nanoparticle uptake the viability of macrophages is not significantly different from control conditions. Taken together these cellular assays demonstrate that AR NPs are phagocytized by macrophages and uptake of AR nanoparticles does not interfere with macrophage viability.

## Discussion

The use of nanotechnology has exploded in the recent years. Nanoparticles were initially developed as carriers for vaccines and cancer chemotherapy agents [25-27]. The use of nanotechnology as a drug delivery system has mainly been investigated for the treatment of malignancies. Nanoparticles can concentrate preferentially in tumor masses, inflammatory sites, and infectious sites by utilization of enhanced permeability and retention (EPR)effect on the vasculature [28]. Modifying oncologic drugs into nanoparticles and delivering the drug to the malignant tissue has resulted in significant preliminary results in animal models [29]. Indeed, this is where the majority of research has been focused [30].

Other investigators have been able to fabricate single antiretroviral drugs into a nanoparticle delivery system [14-20]. However, the use of a single antiretroviral drug in the treatment of HIV-1 only results in development of resistant strains and treatment failures. Combination drugs are currently the standard of practice for HIV-1 therapeutics. Our results demonstrate that three drugs can be incorporated into a single nanoparticle for drug delivery.

The results of cellular assays show that macrophages engulf these particles. This is advantageous, as HIV-1 requires host DNA replication for survival. Providing a means to get significant drug concentrations intracellular would inhibit the replication of HIV-1 in the reticuloendothelial system (RES) where macrophages migrate. Further studies are on going to determine this. Additionally, preliminary MTT assay results show that PLGA particles do not produce significant cellular toxicity. This is also advantageous for development of these nanoparticles as a drug delivery modality for human use. Taken together, our data show that these inert particles are taken up by the macrophages and have a sustained-release profile. The results demonstrate the day 28 levels of AR drugs were ≥ 0.9 μg within MDMs. This level is above the inhibitory concentration (IC_50_) for wild-type virus for each of AR drugs incorporated into these NPs. The IC_50 _for each of the drugs against wild-type virus has been reported to be < 100 ng/ml. While further studies are necessary, this delivery system may have potential advantages.

Viral reservoirs within the body have prevented total eradication of HIV-1 with successful AR [31]. A number of studies have demonstrated persistent, low level HIV-1 replication in patients receiving oral highly active antiretroviral therapy (HAART) that renders them aviremic [32-34]. These studies provide evidence that continued viral replication occurs in lymphoid reservoirs. Our studies show PLGA AR nanoparticles within the cytoplasm of macrophages. The PLGA AR nanoparticles are phagocytosized by macrophages and these cells could deliver high AR levels to lymphoid reservoirs and could positively affect persistent, low level viral replication. This could prevent the development of mutant HIV-1 virions to AR drugs. Further research is necessary to determine the concentration of AR drugs in these lymphoid reservoirs as well as gut-associated lymphoid tissue (GALT) [35].

The results of these experiments demonstrate for the first time that combination antiretroviral drugs can be loaded efficiently into a nanoparticle drug delivery system. Our data show that sustained drug release over the course of 28 days is possible. The goal of drug delivery systems is cellular uptake and release with no cytotoxicity. Indeed, this drug delivery system is advantageous as it could preclude the need for daily administration of oral drugs to maintain active concentrations in HIV-1 tissues with lower total amount of drug exposure. Therefore, this delivery method may be useful for patients that are nonadherent to orally administered HAART and may offer other patients treatment options. Additionally, since the majority of the AR drugs are incorporated into the NP, the amount of AR NPs necessary as dosage strength may be lower. This could potentially reduce side effects that patients currently have to tolerate for successful adherence. Finally, if patients received ~100% of their AR drugs, the development of resistance would slow and the efficacy and durability of AR drug therapeutics would be enhanced [36-38]. Of note, an investigational non-nucleotide reverse transcriptase inhibitor (NNRTI) capable of once every 8 weeks administration has been recently published showing that sustained delivery of antiretrovirals may be utilized clinically [39]. Further studies are necessary to produce reliable data regarding the pharmacology and efficacy of this delivery system. These data provides further evidence that sustained release of multiple AR drugs from a nanoparticle drug delivery system present a viable option for treatment of HIV-1.

## Conclusion

The results of these experiments demonstrate that PLGA polymer can be used to fabricate nanoparticles for combination AR to develop a drug delivery system that can be used for IV administration to patients who are nonadherent to their therapy. Furthermore, this delivery system has prolonged release of combination AR for 28 days. The particles penetrate macrophages and do not cause toxicity to these cells by MTT assay. Further study is necessary to determine the drug concentrations in lymphoid tissue. This could be a promising new delivery system for the management of HIV-1 infected patients.

## Abbreviations

PLGA: poly-(lactic-co-glycolic acid); HIV-1: human immunodeficiency virus type 1; AR: antiretroviral; PBMCs: peripheral blood mononuclear cells; RTV: ritonavir; LPV: lopinavir; EFV: efavirenz; NP: nanoparticle; PBS: phosphate buffered saline; MDM: monocytes-derived macrophage; MTT: 3-(4,5-dimethylthiazol-2-yl)-2,5-diphenyltetrazolium bromide; ddH_2_O: double distilled water; PVA: polyvinyl alcohol; SEM: scanning electron microscopy; TEM: transmission electron microscopy; HPLC: high-pressure liquid chromatography; M-CSF: recombinant human macrophage colony stimulating factor; DNA: deoxyribonucleic acid; RES: reticuloendothelial system; HAART: highly active antiretroviral therapy; GALT: gut-associated lymphoid tissue.

## Competing interests

CJD is currently applying for a patent relating to the content of this manuscript. None of the other authors have competing interests for this manuscript. Parts of this manuscript were presented as a poster (F-128) at the 2008 Conference on Retrovirus and Opportunistic Infections (CROI), Boston, MA Feb 3-6.

## Authors' contributions

CJD was responsible for study design and overall data collection, analysis, and writing manuscript. TB was responsible for carrying out experiments. KC was responsible for experimental design and editing manuscript. AS preformed MTT experiments, fluorescent experiments, writing and editing manuscript. AS was responsible for editing manuscript and expert opinion. AKD was responsible for editing manuscript and expert opinion. All authors have read and approved the final manuscript.

## Pre-publication history

The pre-publication history for this paper can be accessed here:

http://www.biomedcentral.com/1471-2334/9/198/prepub

## References

[B1] Piot P Bartos M Ghys PD Walker N Schwartlander B The global impact of HIV/AIDS Nature 2001 410 968 973 10.1038/35073639 11309626

[B2] Chen RY Westfall AO Raper JL Cloud GA Chatham AK Acosta EP Immunologic and virologic consequences of temporary antiretroviral treatment interruption in clinical practice AIDS Trd Hum Retroviruses 2002 18 900 916 10.1089/088922202760265588 12230934

[B3] Chulamokha L DeSimone JA Ponerantz RJ Antiretroviral therapy in the developing world J Neurovirol 2005 11 76 80 10.1080/13550280590900436 15960241

[B4] Fellay J Boubaker K Ledergerber B Bernasconi E Furrer H Battegay M Prevalence of adverse events associated with potent antiretroviral treatment: Swiss HIV Cohort Study Lancet 2001 358 1322 1327 10.1016/S0140-6736(01)06413-3 11684213

[B5] Gartner S Markovits P Markovitz DM Kaplan MH Gallo RC Popovic M The role of mononuclear phagocytes in HTLV-III/LAV infection Science 1986 233 215 219 10.1126/science.3014648 3014648

[B6] Kuhnel H von Briesen H Dietrich U Meichsner C Adamski M Kreutz R Molecular cloning of two Wet African human immunodeficiency virus type 2 isolates that replicate well in macrophages: a Gambian isolate, from a patient with neurologic acquired immunodeficiency syndrome, and a highly divergent Ghanian isolate Proc Natl Acad Sci USA 1989 86 2382 2387 10.1073/pnas.86.7.2383 PMC286917 2467304

[B7] Nicholson JK Cross GD Callaway CS McDougal JS In vitro infection of human monocytes with human T lymphotropic virus type III/lymphadenopathy-associated virus (HTLV-III/LAV) J Immunol 1986 137 323 329 3011909

[B8] von Briesen J Andreesen R Esser R Brugger W Meichsner C Becker K Rubsamen-Waigmann H Infection of monocytes/macrophages by HIV in vitro Res Virol 1990 141 225 231 10.1016/0923-2516(90)90025-E 1693221

[B9] McGann K Collman AR Kolson DL Gonzalez-Scarano F Coukos G Coutifaris C Human immunodeficiency virus type 1 causes productive infection of macrophages in primary placental cell cultures J Infect Dis 1994 169 746 753 8133087 10.1093/infdis/169.4.746

[B10] Milman G Sharma O Mechanisms of HIV/SIV mucosal transmission AIDS Res Hum Retroviruses 1994 10 1305 1312 10.1089/aid.1994.10.1305 7848686

[B11] Kingsley JD Dou H Morehead J Rabinow B Gendelman HE Destache CJ Nanotechnology: a focus on nanoparticles as a drug delivery system J Neuroimmun Pharmacol 2006 1 340 350 10.1007/s11481-006-9032-4 18040810

[B12] Beck PH Kreuter J Muller WEG Schatton W Improved peroral delivery of avoral with polybutylcyanoacrylate nanoparticles Eur J Pharm Biopharm 1994 40 134 137

[B13] Kreuter J Kreuter J Nanoparticles Colloidal Drug Delivery Systems 1994 New York: Marcel Dekker, Inc. 219 342

[B14] Dou H Destache CJ Morehead JR Mosley RL Boska MD Kingsley J Development of a macrophage-based nanoparticle platform for anti-retroviral drug delivery Blood 2006 108 2827 2835 10.1182/blood-2006-03-012534 16809617 PMC1895582

[B15] Dou H Morehead J Destache CJ Kingsley JD Skyakhtenko L Zhou U Laboratory investigations for the morphologic, pharmacokinetic, and anti-retroviral properties of indinavir nanoparticles in human monocytes-derived macrophages Virology 2007 358 148 158 10.1016/j.virol.2006.08.012 16997345

[B16] Gorantla S Dou H Boska M Destache CJ Nelson JA Poluektova L Quantitative magnetic resonance and SPECT imaging for macrophage tissue migration and nanoformulated drug delivery J Leukoc Biol 2006 80 1165 1174 10.1189/jlb.0206110 16908517

[B17] Gagne J-F Desormeaux A Perron Tremblay MJ Bergeron MG Targeted delivery of indinavir to HIV-1 primary reservoirs with immunoliposomes Biochem Biophy Acta 2002 1558 198 210 10.1016/S0005-2736(01)00432-1 11779569

[B18] Bender AR von Briesen H Kreuter J Duncan IB Rubsamen-Waigmann H Efficiency of nanoparticles as a carrier system for antiretroviral agents in human immunodeficiency virus-infected human monocytes/macrophages in vitro Antimicrob Agents Chemother 1996 40 1467 1471 8726020 10.1128/aac.40.6.1467 PMC163350

[B19] Kuo Y-C Loading efficiency of stavudine on polybutylcyanoacrylate and methmethacrylate-sulfopropylmethacrylate copolymer nanoparticles Int J Pharmaceut 2005 290 161 172 10.1016/j.ijpharm.2004.11.025 15664142

[B20] Shah LK Amiji MM Intracellular delivery of saquinavir in biodegradable polymeric nanoparticles for HIV/AIDS Pharm Res 2006 23 2638 2645 10.1007/s11095-006-9101-7 16969696

[B21] Weller DR Brundage RC Balfour J H H Vezina HE An isocratic liquid chromatography method for determining HIV non-nucleoside reverse transcriptase inhibitor and protease inhibitor concentrations in human plasma J Chromatrograph B Analyt Technol Biomed Life Sci 2007 848 369 373 10.1016/j.jchromb.2006.10.022 17081812

[B22] Gendelman HE Orenstein JM Martin MA Ferrua C Mitra R Phipps T Efficient isolation and propagation of human immunodeficiency virus on recombinant colony-stimulating factor-1-treated monocytes J Exp Med 1988 167 1428 1441 10.1084/jem.167.4.1428 3258626 PMC2188914

[B23] Denizot F Lang R Rapid colorimetric assay for cell growth and survival. Modifications to the tetrazolium dye procedure giving improved sensitivity and reliability J Immunol Methods 1986 89 271 277 10.1016/0022-1759(86)90368-6 3486233

[B24] Hansen MN Nielsen SE Berg K Re-examination and further development of a precise and rapid dye method for measuring cell growth/cell kill J Immunol Methods 1989 119 203 210 10.1016/0022-1759(89)90397-9 2470825

[B25] Couvreur P Kante B Crislain L Roland M Speiser P Toxicity of polyalkylcyanoacrylate nanoparticles II: doxorubicin-loaded nanoparticles J Pharm Sci 1982 71 790 792 10.1002/jps.2600710717 7120064

[B26] Beck P Kreuter J Reszka R Fichtner I Influence of polybutylcyanoacrylate nanoparticles and liposomes on the efficacy and toxicity of anticancer drug mitoxantrone in murine tumour models J Microencapsul 1993 10 101 114 10.3109/02652049309015316 8445503

[B27] Conway MA Madrigal-Estebas L McClean S Brayden DJ Mills KH Protection against *Bordetella pertussis *infection following parenteral or oral immunization with antigens entrapped in biodegradable particles: effect of formulation and route of immunization on induction of Th_1 _and Th_2 _cells Vaccine 2001 19 1940 1950 10.1016/S0264-410X(00)00433-3 11228364

[B28] Shenoy D Little S Langer R Amiji M Poly(ethylene oxide)-modified poly(β-amino ester) nanoparticles as a pH-sensitive system for tumor-targeted delivery of hydrophobic drugs: Part 2. In vivo distribution and tumor localization studies Pharm Res 2005 22 2107 2114 10.1007/s11095-005-8343-0 16254763 PMC1350958

[B29] Sengupta S Eavarone D Capila I Zhao G Kiziltepe T Sasisekharan R Temporal targeting of tumour cells and neovasculature with a nanoscale delivery system Nature 2005 436 568 572 10.1038/nature03794 16049491

[B30] Brannon-Pepas L Blanchette JO Nanoparticle and targeted systems for cancer therapy Adv Drug Del Rev 2004 56 1649 1659 10.1016/j.addr.2004.02.014 15350294

[B31] Chun TW Davey RT Engel D Lane HC Fauci AS Re-emergence of HIV after stopping therapy Nature 1999 401 874 875 10.1038/44755 10553903

[B32] Zhang L Ramratnam B Tenner-Racz K He Y Vesanen M Lewin S Quantifying residual HIV-1 replication in patients receiving combination antiretroviral therapy N Engl J Med 1999 340 1605 1613 10.1056/NEJM199905273402101 10341272

[B33] Natarajan V Bosche M Metcalf JA WArd DJ Lane HC Kovacs JA HIV-1 replication in patients with undetectable plasma virus receiving HAART Lancet 1999 353 119 120 10.1016/S0140-6736(05)76156-0 10023903

[B34] Ramratnam B Ribeiro R He T Chung C Simon B Vanderhoeven J Intensification of antiretroviral therapy accelerates the decay of the HIV-1 latent reservoir and decreases, but does not eliminate, ongoing virus replication J Acquire Immune Defic Syndr 2004 35 33 37 10.1097/00126334-200401010-00004 14707789

[B35] Chun T-W Nickle DC Justement JS Meyers JH Roby G Hallahan CW Persistence of HIV in gut-associated lymphoid tissue despite long-term antiretroviral therapy J Infect Dis 2008 197 714 720 10.1086/527324 18260759

[B36] Palepu A Tyndall MW Joy R Kerr T Wood E Press N Antiretroviral adherence and HIV treatment outcomes among HIV/HCV co-infected injection drug users: the role of methadone maintenance therapy Drug Alcohol Depend 2006 84 188 194 10.1016/j.drugalcdep.2006.02.003 16542797

[B37] Farmer P Leandre F Mukherjee J Gupta R Tarter L Kim JY Community-based treatment of advanced HIV disease: introducing DOT-HAART (directly observed therapy with highly active antiretroviral therapy) Bulletin World Health Org 2001 79 1145 1151 PMC2566712 11799447

[B38] Rockstron J Dejesus E Donatacci L Wat C Bertasso A Labriola-Tompkins E Adherence to Enfuvirtide and its impact on treatment efficacy AIDS Res Hum Retroviruses 2008 2 141 148 10.1089/aid.2006.0231 18240965

[B39] Baert L van't Klooster G Dries W Francois M Wouters A Basstanie E Development of a long-acting injectable formulation with nanoparticles of rilpivirine (TMC278) for HIV treatment Eur J Pharm Biopharm 2009 72 502 508 Epub 2009 Mar 2027. 10.1016/j.ejpb.2009.03.006 19328850

